# Whey Protein Isolate-Encapsulated Astaxanthin Nanoemulsion More Effectively Mitigates Skeletal Muscle Atrophy in Dexamethasone-Induced Mice

**DOI:** 10.3390/nu17050750

**Published:** 2025-02-20

**Authors:** Yuchen Huan, Han Yue, Yanli Song, Wenmei Zhang, Biqian Wei, Qingjuan Tang

**Affiliations:** 1 College of Food Science and Engineering, Ocean University of China, Qingdao 266400, China; huanyuchen79@outlook.com (Y.H.); yhyh_000219@163.com (H.Y.); wenmei20141221@163.com (W.Z.); weibiq00@163.com (B.W.); 2 Department of Emergency, Tongji Hospital, Tongji University School of Medicine, Shanghai 200065, China; songyanli@tongji.edu.cn

**Keywords:** astaxanthin, dexamethasone, whey protein isolate, skeletal muscle atrophy, nanoemulsion

## Abstract

Background: Skeletal muscle, as the largest organ in the body and the main protein pool, is crucial for various physiological processes, but atrophy of skeletal muscle can result from glucocorticoids, including dexamethasone, or from aging. Astaxanthin (AST) is a ketocarotenoid with a variety of physiological activities. However, the clinical application of AST is hampered by its strong hydrophobicity, intense off-flavors, and susceptibility to oxidation. Methods: In this study, we prepared whey protein isolate (WPI)-encapsulated AST nanoemulsion (WPI-AST, W-A) and investigated its alleviating effects on dexamethasone-induced skeletal muscle atrophy. Results: The optimal concentration of astaxanthin was determined to be 30 mg/mL with an oil/water ratio of 1:5. The W-A was a typical oil-in-water (O/W) emulsion with a particle size of about 110 nm. The bioaccessibility of astaxanthin was significantly improved, with the off-flavors of astaxanthin effectively masked. After oral administration, the W-A further ameliorated skeletal muscle atrophy by inhibiting skeletal muscle catabolism, promoting skeletal muscle production, and inhibiting mitochondrial autophagy compared with the same dose of WPI and AST. In addition to this, the W-A further improved the glycometabolism of skeletal muscle by reducing the expression of Foxo3 and increasing the expression of PGC-1α. Conclusions: In conclusion, the W-A nanoemulsion demonstrated good therapeutic value in alleviating skeletal muscle atrophy.

## 1. Introduction

Skeletal muscle, as the body’s largest organ and a major protein reservoir, plays a pivotal role not only in the control of locomotion [1], but also in a variety of physiological processes, including energy homeostasis, respiration, and regulation of nutrient metabolism [2]. Skeletal muscle atrophy has been defined as a reduction in muscle weight and function, and it is often caused by a variety of factors, such as glucocorticoid (GC) administration, aging, denervation, and pathological conditions, which can affect overall metabolism. Although a number of nutritional and dietary approaches exist to prevent or treat atrophy [3], including protein and β-hydroxy β-methylbutyrate (HMB), no effective therapies have been approved for clinical use [4]. Although higher dietary protein is beneficial for atrophy, there are still many barriers to reaching this dietary goal, so more diverse treatments are needed [5].

Studies have found that low dietary antioxidant intake in the elderly is associated with lower muscle strength [6]. There was also a negative correlation between oxidative stress status and skeletal muscle strength in the US population [7]. All this evidence indicated that dietary antioxidants appeared to be critical for maintaining muscle health. Astaxanthin, a ketocarotenoid with high antioxidant capacity, possesses such as anti-inflammatory, anti-aging, and immune-enhancing activities [8]. It has been reported to alleviate skeletal muscle atrophy as well as enhance athletic performance through mitochondrial adaptations [9,10,11]. However, the clinical application of AST is hampered by its strong hydrophobicity, intense off-flavors, and susceptibility to oxidation [12].

To address this unmet medical need, practical and safe oral drug delivery strategies for AST need to be developed. Proteins are promising nanocarriers due to their non-toxic and biodegradable properties [13,14]. Whey protein isolate (WPI), which mainly consists of β-lactoglobulin (β-Lg) (about 70%) and α-lactalbumin (α-Lac) (about 25%), is an edible, cost-effective protein and one of the most accepted natural emulsifiers [15,16]. It has good safety and market acceptance.

In addition to this, WPI has been found to be the best supplement to combat atrophy in elderly people undergoing resistance training (RT) [17]. Astaxanthin-loaded WPI emulsions have been reported, and it was found that WPI could be used as an optimal carrier for encapsulated astaxanthin as an oral nanocarrier with potential clinical applicability [15]. Astaxanthin encapsulated by WPI could transport through Caco-2 cells, which demonstrated good bioavailability [18]. In addition, the use of astaxanthin with modified WPI and with other polysaccharides has shown enhanced physiological activity and promising applications in food processing. Although current research has focused on the use of WPI in astaxanthin products, the complexity of the preparation materials and the use of organic solvents have led to many limitations in practical applications [19,20]. Therefore, we hope that by adjusting the parameters of the preparation method, relying on the physical methods of homogenization and ultrasound to obtain a more economical, safe, and stable astaxanthin emulsion. By combining the synergistic potential of the two, the promising application of this system for the alleviation of skeletal muscle atrophy was researched. Therefore, in this study, O/W type nanoemulsion was prepared by a simple method to further investigate the ameliorative effect of WPI-based astaxanthin nanoemulsion on skeletal muscle atrophy.

## 2. Materials and Methods

### 2.1. Materials

The whey protein isolate (WPI), purchased from Fonterra Co-operetive Group (Auckland, New Zealand), has a protein content of 93% (*w*/*w*). The astaxanthin was derived from *Haematococcus pluvialis* (*H. pluvialis*) and obtained from Yunnan Alphy Biotech Co., Ltd. (Yunnan, China). The astaxanthin concentration was 10% (*w*/*w*) and consisted of astaxanthin monoesters, astaxanthin diesters, and free astaxanthin (71%, 28%, and <1%, respectively) [21]. Salivary amylase and pepsin were purchased from Shanghai Yuanye Biotechnology Co., Ltd. (Shanghai, China). Trypsin and porcine bile salt were purchased from G-CLONE Biotechnology Co., Ltd. (Beijing, China). Oxymetholone was purchased from Shanghai Jizi Biochemical Technology Co., Ltd. (Shanghai, China). Dexamethasone was purchased from Solarbio Technology Co., Ltd. (Beijing, China). Olive oil was purchased from a local supermarket. The rest of the reagents were of analytical grade and obtained from Sinopharm Chemical Reagent Co., Ltd. (Shanghai, China).

### 2.2. Preparation of Emulsion

Sample preparation followed previous methods [22], where a solution of WPI at a concentration of 3% (*w*/*w*) was prepared by dissolving into ultrapure water, stirring for 1 h at room temperature, and refrigerating overnight at 4 °C. Different concentrations of astaxanthin were dissolved in olive oil and stirred overnight away from light. Astaxanthin was mixed into the WPI solution in specific proportions and homogenized using a homogenizer (T25, IKA, Staufen, Germany) at 10,000 rpm for 3 min to obtain a crude emulsion. Then, the WPI-AST emulsion (W-A) was immediately prepared by ultrasonic homogenization (JY92-IIN, SCIENTZ, Ningbo, China) in an ice-water bath for 5 min (with a 2 s rest every 3 s of work) at an ultrasonic power of 390 W. To explore the appropriate concentration of astaxanthin, the oil/water ratio was fixed at 1:9 (*v*/*v*), and the astaxanthin concentration in the oil phase was adjusted to 0 mg/mL, 15 mg/mL, 30 mg/mL, and 60 mg/mL. To explore the appropriate oil/water ratio, the astaxanthin concentration in the oil was fixed at 30 mg/mL, and the oil/water ratio (*v*/*v*) was adjusted to 1:9, 2:13, 1:5, 1:4, and 1:2, respectively.

### 2.3. Particle Size and Zeta Potential Determination

The particle size distribution, polydispersity index (PDI), and zeta potential of the emulsions were determined using a Malvern laser particle sizer (Zetasizer Nano-ZS90, Malvern, UK) at 25 ± 1 °C. The measurements were performed by diluting the emulsion 20 times with an equilibration time of 120 s [23]. Each sample was measured three times.

### 2.4. Observation of Microscopic Morphology

Referring to the previous method [24,25], a drop of emulsion was placed on a grooved slide and gently covered with a coverslip, and the morphology and size of the emulsion droplets were observed under a fluorescence inverted microscope (Nikon Ni-E, Tokyo, Japan). The emulsion was diluted 50 times and placed on a copper grid, and the morphology of the emulsion was observed under an accelerating voltage of 200 kV using a transmission electron microscope (TEM) (JEM2100F, JEOL Ltd., Tokyo, Japan).

### 2.5. Measurement of Encapsulation Efficiency of Emulsion

According to reported methods [26], the emulsion was diluted 200 times with DMSO to form a clear and transparent solution. A total of 200 μL of the sample was taken in a 96-well plate, and the absorbance value was measured at 474 nm using a microplate reader (SPARK 10M, Tecan, Männedorf, Switzerland). The emulsion without added astaxanthin was used as a control. The astaxanthin content was calculated from the pre-established astaxanthin standard curve (y = 9.6349x + 0.0481, R^2^ = 0.998). The encapsulation efficiency was calculated according to the following formula:

(1) $$\mathrm{EE}\%=\left(\frac{Encapsulated\;ASX\;content}{total\;ASX\;input}\right)\times 100\%$$

### 2.6. In Vitro Digestion Experiment

According to reported methods with slight modifications [27], an in vitro digestion model was constructed to simulate the digestive process of the emulsion in the oral cavity, stomach, and small intestine.

Oral digestion stage: The simulated oral fluid contained 0.896 mg/mL of KCl, 0.6 mg/mL of α-amylase, and 0.298 mg/mL of NaCl. A total of 5 mL of the emulsion was taken, mixed with 5 mL of oral digestive fluid, and oscillated for 5 min at 100 rpm in a water bath at 37 °C. The emulsion was then digested in the stomach and small intestine.

Gastric digestion stage: The simulated gastric fluid contained 3.2 mg/mL of pepsin and 2 mg/mL of NaCl. The pH of the gastric fluid was adjusted to 3.0 with 0.7% (*v*/*v*) HCl. The whole mixture (10 mL) after oral digestion was mixed with 30 mL of gastric digestive fluid and oscillated for 2 h at 100 rpm in a water bath at 37 °C.

Intestinal digestion stage: The simulated intestinal fluid contained 6.8 mg/mL of K_2_HPO_4_, 8.8 mg/mL of NaCl, 12 mg/mL of porcine bile salts, and 3.2 mg/mL of trypsin. The pH of the gastro-digested mixture was adjusted to 7.0 with 1.0 M NaOH. All of the gastro-digested mixture (40 mL) was mixed with 80 mL of the entero-digested mixture and oscillated for 2 h at 100 rpm in a water bath at 37 °C.

The digested samples from the oral cavity, stomach, and small intestine were taken, their micro-morphology was observed, and the average particle size and zeta potential were determined. The digested end products were transferred to a centrifuge tube and centrifuged at 10,000 rpm for 30 min at 4 °C. After centrifugation, the middle micellar layer was taken and dissolved in DMSO to determine the astaxanthin content. The bioaccessibility of astaxanthin was calculated according to the following equation:

(2) $$\mathrm{Bioaccessibility}(\%)=\left(\frac{C_{micelle\;layer}}{C_{initial\;emulsion}}\right)\times 100\%$$

where C_micelle layer_ is the astaxanthin content in the micelle layer, and C_initial emulsion_ is the astaxanthin content in the emulsion.

### 2.7. GC-IMS Analysis

Referring to the previous method [28], a 2 mL sample was taken into a 20 mL headspace injection vial, and the volatile flavor substances were determined by gas chromatography-ion mobility spectrometry (GC-IMS) (G.A.S. Technology Co., Ltd., Dortmund, Germany). The samples were incubated at 40 °C for 20 min and injected headspace. A DB-5MS UI non-polar meteorological column was used. Nitrogen was used as the carrier gas, the temperature of the injection port was 40 °C, the flow rate of the column was 2 mL/min, and the flow rate of the drift gas was 150 mL/min. The injection program was set as follows: 2 mL/min held for 10 min, 10 mL/min held for 5 min, 100 mL/min held for 5 min, and finally increased to 150 mL/min held for 10 min, and the total running time was 30 min. The flavor compounds in the samples were determined by comparing the detected compounds with the retention index (IR) and relative drift time (RIP) of the standards in the database.

### 2.8. Effect of the W-A on Skeletal Muscle Atrophy in Mice

#### 2.8.1. Animal Experiment

Animal experiments were performed in accordance with previous studies with modifications [11]. Healthy 8-week-old male C57BL/6J mice (*n* = 48, 20 ± 2 g) were purchased from Vital River Laboratory Animal Technology Co., Ltd. (Beijing, China). All animals were housed in a specific pathogen-free (SPF) animal center with constant temperature (22 ± 0.5 °C) and humidity (50 ± 5% relative humidity) under a 12 h/12 h light/dark cycle. This experiment was approved by the Laboratory Animal Ethics Committee of the Ocean University of China (Permission No. SPXY2024051601). The mice were randomly divided into 6 groups (*n* = 8 in each group) after 7 days of acclimatization feeding: (1) normal group (Con), pure water gavage; (2) model group (Sar), pure water gavage; (3) positive drug group (Oxy), Oxymetholone (50 mg/kg/d) gavage; (4) whey protein isolate group (WPI), whey protein (360 mg/kg/d) gavage; (5) astaxanthin group (Ast), astaxanthin (60 mg/kg/d) gavage; (6) WPI-astaxanthin emulsion group (W-A), whey protein–astaxanthin emulsion (12 mL/kg/d, equivalent to 360 mg/kg/d WPI and 60 mg/kg/d astaxanthin) gavage. The skeletal muscle atrophy model was established by intraperitoneal injection of dexamethasone (5 mg/kg/d) in the remaining groups, except for the normal group, which was injected intraperitoneally with saline. The treatment was continued for 18 days, and the body weight and food intake of the mice were recorded every other day. On the last day of the experiment, the mice were euthanized (Cervical dislocation was performed after anesthesia via intraperitoneal injection of pentobarbital sodium salt). The skeletal muscles (gastrocnemius, tibialis anterior, and soleus) and epididymal fat were weighed and stored at −80 °C until the next analysis.

#### 2.8.2. Histopathological Evaluation

The gastrocnemius muscle tissue was fixed with 4% paraformaldehyde, dehydrated, and embedded in paraffin, and sections were made and stained with hematoxylin and eosin (H&E) (Servicebio, Wuhan, China). The histomorphology of the gastrocnemius muscle was observed under a light microscope. The cross-sectional area of muscle fibers was analyzed using ImageJ 1.54f software [11].

#### 2.8.3. Western Blotting Experiment

The gastrocnemius muscle tissue was immersed in RIPA lysate (Solarbio, Beijing, China), ground, and centrifuged to extract skeletal muscle proteins. The protein concentration was determined using a BCA protein quantification kit (Epizyme Biotech, Shanghai, China) and adjusted to 5 μg/μL for subsequent experiments. A total of 25 mg protein from each group was separated by SDS-PAGE electrophoresis and then transferred to a polyvinylidene tetrafluoride (PVDF) membrane. The membranes were placed in 5% skim milk powder and closed for 2 h at room temperature. Primary antibodies were added overnight at 4 °C. Myod1 (1:1000), MuRF1 (1:1000), BNIP3 (1:1000), Lc3B (1:500), and Foxo3 (1:1000) were purchased from ABclonal (Wuhan, China). PGC-1α (1:1000) and GAPDH (1:2000) were purchased from ZEN BIO (Chengdu, China) and Servicebio (Wuhan, China), respectively. The PVDF membranes were washed with TBST and then immersed in HRP-labeled goat anti-rabbit IgG secondary antibody for 2 h at room temperature. The PVDF membranes were washed again and imaged with a chemiluminescence imaging instrument (Tanon-5200, Shanghia, China). The bands were analyzed using ImageJ software [11].

#### 2.8.4. Biochemical Assays

The levels of lactate, glycogen, and succinate dehydrogenase in skeletal muscle and serum were determined according to the instructions of the kit (Nanjing Jianjieng Bioengineering Institute, Nanjing, China) [11].

#### 2.8.5. RNA Extraction and RT-qPCR

Total RNA was extracted from gastrocnemius muscle using Trizol reagent [11]. RNA was reverse transcribed to cDNA using All-In-One 5X RT MasterMix (Abmgood, Shanghai, China) according to the instruction manual. The cDNA was diluted appropriately. RT-qPCR analysis was performed using the BlasTaq™ 2X qPCR MasterMix kit (Abmgood, Shanghai, China) according to the instructions. Primers are shown in Table 1, which were synthesized by Shanghai Sangong Biotechnology (Shanghai, China). The relative expression of each gene was calculated using the 2^−∆∆CT^ method, with GAPDH as an internal reference.

### 2.9. Statistical Analysis

This part was performed using GraphPad Prism 9.5.0 (San Diego, CA, USA) software for data analysis and plotting. Data were expressed as mean ± standard error of mean (mean ± SEM). After Bartlett’s test and Shapiro–Wilk test, statistical analysis was performed by one-way analysis of variance (ANOVA), followed by Dunnett’s post hoc test. *p* < 0.05 indicated a statistically significant difference.

## 3. Results

### 3.1. The Effect of Astaxanthin Concentration on Emulsions

Astaxanthin derived from *Haematococcus pluvialis* (*H. pluvialis*) is usually a viscous solution and often needs to be diluted into olive oil or other oil solvents for use. In a previous study conducted by our group, an AST administration concentration of 60 mg/mL for improving skeletal muscle atrophy was determined [11]. Therefore, the total amount of astaxanthin in the emulsion must be considered in order to achieve the expected use requirements, making it necessary to explore the effect of astaxanthin concentration on the preparation of emulsions.

**Figure 1:**
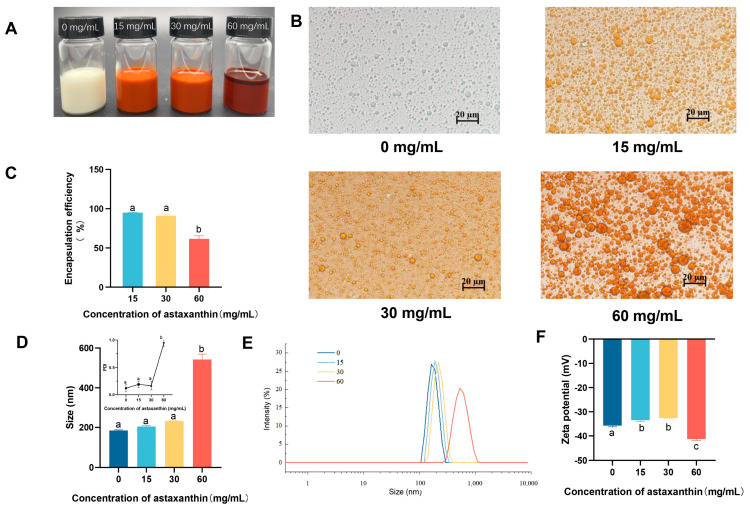
Influence of astaxanthin concentration on emulsions. (**A**) Photograph of emulsions; (**B**) microscope photos; (**C**) encapsulation efficiency of astaxanthin in emulsions; (**D**,**E**) particle size and PDI of emulsions; (**F**) ζ-potential of emulsions with different concentrations of astaxanthin. Data are expressed as mean ± SEM, *n* = 3 for each group. Different letters indicate significant differences.

Three astaxanthin concentrations of 15, 30, and 60 mg/mL were selected for the preparation of emulsions at an oil/water ratio of 1:9. The emulsion added with astaxanthin had an overall orange-red color, but at a concentration of 60 mg/mL, it showed astaxanthin precipitation (Figure 1A). Concentrations of AST at 15 and 30 mg/mL exhibited higher encapsulation rates of 94.94 ± 0.65% and 91.07 ± 3.17%, respectively, compared with that at 60 mg/mL (61.62 ± 5.32%) (Figure 1C). It showed that the groups of 0, 15, and 30 mg/mL had dispersed particles with small sizes, whereas the 60 mg/mL group had larger particles (d = 542.27 ± 38.19 nm) with particle adhesion (Figure 1B,D). For PDI, except for the sample with astaxanthin concentration of 60 mg/mL (0.94 ± 0.08), which had a high PDI, the other samples exhibited low PDI values (≤0.3) (Figure 1D). With the increase of astaxanthin concentration, the absolute value of the potential gradually decreased, and the electrostatic interaction between droplets became poor. However, when the astaxanthin concentration was 60 mg/mL, the absolute value of the potential became larger (Figure 1E,F). This may be due to the increased astaxanthin causing the oil phase to be more viscous and some emulsion to break down, and there being more forces between the oil molecules. Therefore, an astaxanthin concentration of 30 mg/mL was selected for subsequent experiments.

### 3.2. The Effect of Oil/Water Ratio on Emulsions

**Figure 2:**
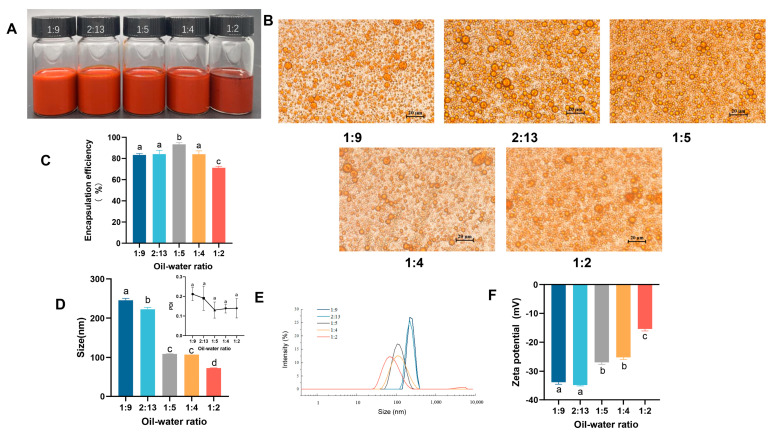
Influence of the oil/water ratio on emulsions. (**A**) Photograph of emulsions; (**B**) microscope photos; (**C**) encapsulation efficiency of astaxanthin in emulsions; (**D**,**E**) particle size and PDI of emulsions; (**F**) ζ-potential of emulsions with different concentrations of astaxanthin. Data are expressed as mean ± SEM, *n* = 3 for each group. Different letters indicate significant differences.

The effect of the oil/water ratio on emulsion preparation was then examined. Five ratios were selected for analysis at an astaxanthin concentration of 30 mg/mL. As shown in the photos, all the emulsions appeared as a homogeneous orange liquid, with a small amount of astaxanthin precipitating from the sample with an oil/water ratio of 1:2 (Figure 2A). Among them, the oil/water ratio at 1:5 exhibited the best astaxanthin encapsulation rate (93.35 ± 1.29%) (Figure 2C). The emulsion morphology was observed microscopically, and as the oil-water ratio increased, emulsion breakage and oil droplet aggregation occurred, and the 1:9, 2:13, and 1:5 conditions showed smaller and uniform emulsion particles (Figure 2B). The particle size of the emulsion decreased with an increasing oil/water ratio, and emulsions with smaller particles were usually preferred (Figure 2D). All samples exhibited a low PDI (≤0.3), indicating that emulsions at an astaxanthin concentration of 30 mg/mL were monodisperse with a lower tendency for accumulation (Figure 2D) [29]. However, the absolute value of the potential gradually and significantly decreased as the oil/water ratio increased, indicating that the emulsion became unstable (Figure 2F). When the oil/water ratio increased to 1:4 and 1:2, the peaks of particle size distribution became broader, indicating that the particle size was not concentrated, and a second peak appeared at 5000 nm at the oil/water ratio of 1:2 (Figure 2E). Thus, it was hypothesized that when the oil/water ratio was increased to a certain degree, WPI might only encapsulate part of the oil phase, and the remaining oil phase existed in a free state or as large particles, resulting in a smaller but unstable average particle size. The above factors were considered when choosing an oil/water ratio of 1:5 for the preparation of the emulsion.

### 3.3. Properties of the Emulsion

**Figure 3:**
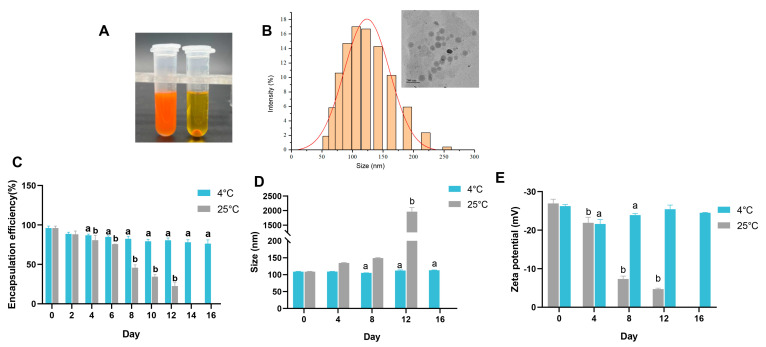
Properties of the emulsion and storage stability. (**A**) Determination of the type of emulsions; (**B**) particle size distribution of the emulsion and TEM images; (**C**) encapsulation efficiency in emulsions during storage; (**D**) changes in the particle size of the emulsion during storage; (**E**) ζ-potential changes of emulsions during storage. Data are expressed as mean ± SEM, *n* = 3 for each group. “a” indicates a significant difference from day 0 at 4 °C, and “b” indicates a significant difference from day 0 at 25 °C.

After determining the optimum preparation process conditions with an astaxanthin concentration of 30 mg/mL and an oil/water ratio of 1:5, the WPI-encapsulated AST nanoemulsion (W-A) was then prepared. The type of the W-A was firstly determined by adding the sample into oil or water, respectively, and it was found that the emulsion dispersed rapidly in water but aggregated in oil, suggesting that the prepared W-A was an O/W emulsion (Figure 3A). The particle size distribution and microscopic morphology of the W-A were observed, and it was found that the emulsion exhibited a homogeneous spherical morphology with a size of about 110 nm (Figure 3B).

### 3.4. Storage Stability, In Vitro Digestive Simulation, and Undesirable Flavor Masking

Suitable storage conditions are important for emulsions in applications. Therefore, storage stability was further explored at 4 and 25 °C, respectively. The encapsulation rate on day 16 was about 79.46% of that on day 0 at 4 °C with an acceptable reduction (Figure 3C), while the particle size and potential remained almost unchanged on day 16 (*p* > 0.05) (Figure 3D,E). However, the encapsulation rate of the W-A decreased sharply at 25 °C for about 8 days (Figure 3C), with the particle size and potential changed (Figure 3D,E). After 12 days of storage at 25 °C, severe breakage of the emulsion was visible to the eyes, and therefore the encapsulation rate, size, and zeta potential were not measured. Overall, the stability of the W-A at 4 °C was better than that at 25 °C.

**Figure 4:**
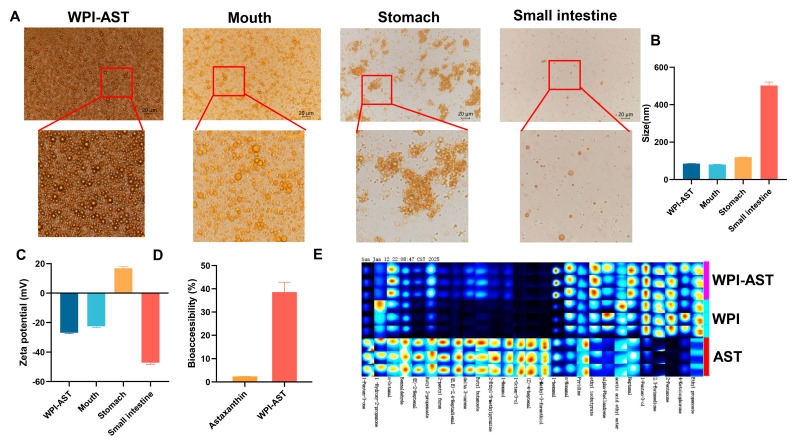
In vitro digestion of the emulsion, flavor characteristics, and bioaccessibility of astaxanthin. (**A**) Microscopic photographs of different digestion stages; (**B**) emulsion particle size changes; (**C**) ζ-potential changes; (**D**) bioaccessibility; (**E**) fingerprinting of volatile flavoring substances. Data are expressed as mean ± SEM, *n* = 3 for each group.

Through microscopy as well as changes in size and zeta potential, the change in the state of the W-A in simulated digestion fluid was examined. The state of the W-A was unchanged in the simulated saliva fluid (SSF), showing a homogeneous state under the microscope, with no change in size or zeta potential. In the SGF, the W-A significantly aggregated and formed large oil droplets due to the destruction of the outer WPI. The zeta potential of the emulsion system became positively charged at this time. It was because the pH at this time was below the isoelectric point of WPI. In the SIF, new mixed micelles were formed, and the droplet diameter became significantly larger due to the presence of porcine bile salts and pancreatic enzymes (Figure 4A–C). The low bioaccessibility of astaxanthin is an important factor limiting its effectiveness. The bioaccessibility of astaxanthin was further examined, and that of astaxanthin in the W-A was increased by 16-fold compared to astaxanthin (Figure 4D). These phenomena were consistent with those previously reported [25].

Astaxanthin’s off-flavors limit its commercial applications. Therefore, odor masking of astaxanthin is of significance for practical application. Twenty-seven kinds of volatile flavor substances were identified by GC-IMS. Astaxanthin mainly exhibits irritating, fishy, greasy, and fruity odors. WPI encapsulation masked unpleasant odors of astaxanthin, especially hexanal, 1-octen-3-ol, and E-2-heptenal. As reported, hexanal and 1-octen-3-ol are the main compounds contributing to the fishy flavor of silver carp products and are produced from lipid oxidation [30,31]. E-2-heptenal is a key odor-active off-flavor contributor in aged pasteurized yogurt (APY) and causes rancidity in virgin olive oils [32,33]. Furthermore, the addition of WPI brought some flavor substances such as 1-penten-3-ol, 2,3-butanedione, and 2-pentanone (Figure 4E). This gave the W-A an overall milky, almondy, fruity, and woody flavor.

### 3.5. Alleviating Effect of the W-A on Dexamethasone-Induced Skeletal Muscle Atrophy in Mice

**Figure 5:**
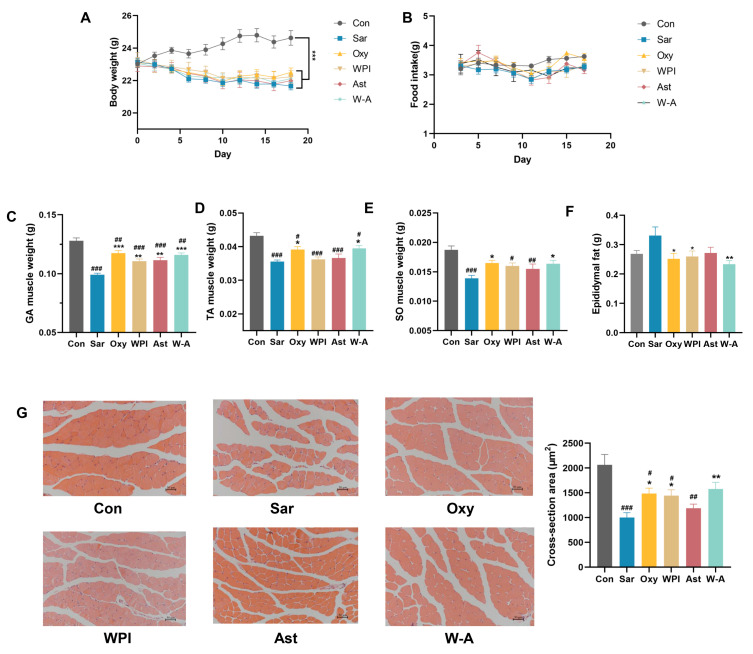
Effect of the W-A on body weight, food intake, and skeletal muscle in mice. (**A**) Weight changes of mice; (**B**) changes in food intake of mice; (**C**) gastrocnemius muscle weight; (**D**) tibialis anterior muscle weight; (**E**) soleus muscle weight; (**F**) epididymal fat weight; (**G**) H&E staining images of skeletal muscle tissues and skeletal muscle cross-sectional area statistics (*n* = 5). Data are expressed as mean ± SEM, *n* = 8 for each group. # *p* < 0.05, ## *p* < 0.01, ### *p* < 0.005, compared with the Con group. * *p* < 0.05, ** *p* < 0.01, *** *p* < 0.005, compared with the Sar group.

Then, we established a model of skeletal muscle atrophy by dexamethasone to investigate the mitigating effect of WPI-astaxanthin emulsion on skeletal muscle atrophy. Dexamethasone induced weight loss in mice; all treatment groups were significantly lower than the normal group (*p* < 0.05), but there was no difference compared with the Sar group (Figure 5A). There was also no significant difference in food intake between the groups, indicating that oxymetholone and interventions did not affect the food intake of the mice (Figure 5B). Skeletal muscle mass could be used to visualize the health status of skeletal muscle. It showed that the W-A group had a better improvement in the mass of the gastrocnemius, tibialis anterior, and soleus muscles than the WPI and AST groups (Figure 5C–E). In addition, dexamethasone led to an increase in epididymal fat weight, and the W-A intervention significantly reduced epididymal fat mass (*p* < 0.01), suggesting that W-A may improve skeletal muscle status by participating in systemic metabolic regulation (Figure 5F).

Skeletal muscle morphology was observed by H&E staining, and the intervention of each subject resulted in tighter skeletal muscle alignment. The WPI and Oxy groups showed significantly increased cross-sectional areas of muscle fibers (*p* < 0.05), with the W-A group showing a better recovery effect (*p* < 0.01) (Figure 5G). Combined with the above apparent indicators, the W-A intervention showed better remission of skeletal muscle atrophy than astaxanthin and WPI intervention alone.

### 3.6. Mechanism of the W-A on Improving Skeletal Muscle Atrophy

**Figure 6:**
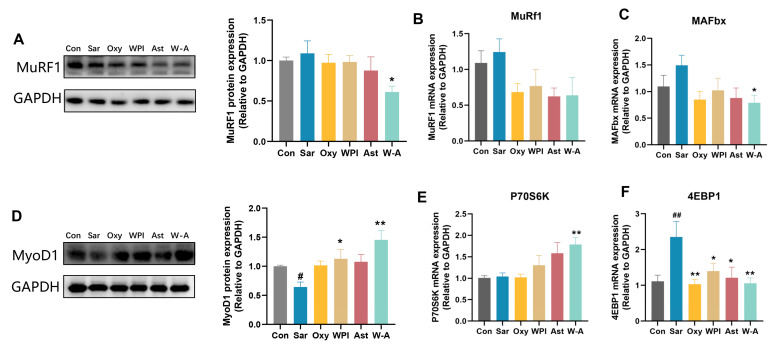
The W-A modulated the synthesis and breakdown of skeletal muscle in mice. (**A**) The protein levels of MuRF1 measured by western blotting; (**B**,**C**) mRNA expression levels of MuRF1 and MAFbx (*n* = 8); (**D**) the protein levels of MyoD1 measured by western blotting; (**E**,**F**) mRNA expression levels of P70S6K and 4EBP1 (*n* = 8). Data are expressed as mean ± SEM, *n* = 3 for each group (western blotting). # *p* < 0.05, ## *p* < 0.01, compared with the Con group. * *p* < 0.05, ** *p* < 0.01, compared with the Sar group.

MuRF1 and MAFbx are ubiquitin proteases that promote the ubiquitinated degradation of skeletal muscle [34]. The expression and transcription levels of MuRF1 were determined by RT-qPCR and western blotting assays. The W-A group showed a significantly reduced protein expression level of MuRF1 compared to the Sar group (*p* < 0.05), with a tendency to reduce the mRNA expression (*p* > 0.05). For MAFbx, the mRNA expression level of MAFbx in the W-A group was significantly lower than that in the Sar group (*p* < 0.05) (Figure 6A–C). The results showed that the W-A further slowed down the degradation of skeletal muscle compared with other treatment groups.

MyoD1 and P70S6K can positively promote skeletal myogenesis, while 4EBP1 inhibits it [35,36]. The protein expression level of MyoD1 was increased in all treatment groups, among which WPI (*p* < 0.05) and the W-A (*p* < 0.01) significantly increased the protein expression level of MyoD1. By measuring transcription levels of P70S6K and 4EBP1, the W-A group demonstrated significant P70S6K recovery (*p* < 0.01) and inhibition of 4EBP1 (*p* < 0.01) (Figure 6D–F). Overall, these results suggested that W-A alleviated skeletal muscle atrophy by further promoting skeletal muscle synthesis and reducing skeletal muscle degradation.

**Figure 7:**
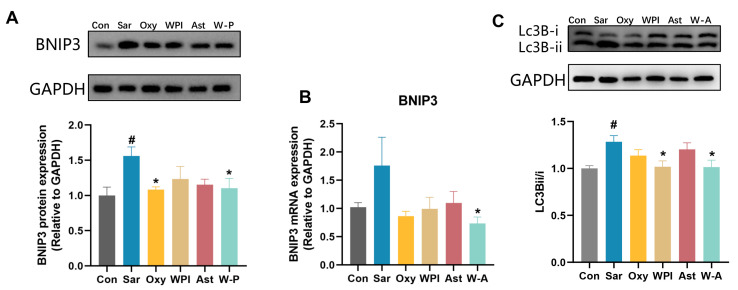
The effect on mitochondrial autophagy in skeletal muscle. (**A**) The protein levels of BNIP3 measured by western blotting; (**B**) mRNA expression levels of BNIP3 (*n* = 8); (**C**) western blotting analysis of Lc3B and ratio of Lc3Bii/Lc3Bi. Data are expressed as mean ± SEM, *n* = 3 for each group (western blotting). # *p* < 0.05, compared with the Con group. * *p* < 0.05, compared with the Sar group.

Mitochondrial damage is an important factor for skeletal muscle atrophy [37]. Activation of autophagy in mitochondria has been reported to contribute to dexamethasone-induced skeletal muscle atrophy [38]. BNIP3, a protein that stimulates programmed cell death and mitochondrial degradation, and LC3B, an autophagy marker, were further tested [10,39]. Dexamethasone markedly increased the expression of BNIP3 and the ratio of Lc3B-ii/i compared with the Con group (*p* < 0.05). WPI and the W-A significantly reduced the ratio of Lc3B-ii/i compared with the Sar group (*p* < 0.05) (Figure 7C). Oxymetholone and the W-A significantly reduced the protein expression of BNIP3 compared with the Sar group (*p* < 0.05), and the W-A had a significant reduction effect on the mRNA expression of BNIP3 (*p* < 0.05) (Figure 7A,B), demonstrating a better ability to inhibit mitochondrial autophagy. These results suggested that the treatment group, including the positive drug, demonstrated the ability to inhibit mitochondrial autophagy, with the W-A group showing better effects.

### 3.7. Effects of the W-A on Glycometabolism in Skeletal Muscle

**Figure 8:**
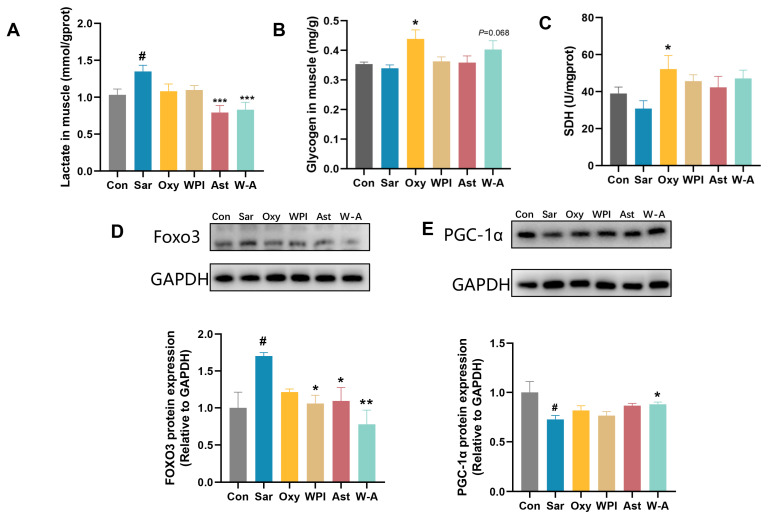
Glycometabolism and expression of proteins in skeletal muscle in mice. (**A**) Lactate level in muscle (*n* = 8); (**B**) glycogen level in skeletal muscle (*n* = 8); (**C**) SDH level in skeletal muscle (*n* = 8); (**D**,**E**) the protein levels of Foxo3 and PGC-1α measured by western blotting (*n* = 3). Data are expressed as mean ± SEM. # *p* < 0.05, compared with the Con group. * *p* < 0.05, ** *p* < 0.01, *** *p* < 0.001, compared with the Sar group.

Dexamethasone treatment leads to disorders of glucolipid metabolism and increases the risk of obesity and diabetes [40]. Dexamethasone resulted in a reduction in skeletal muscle glycogen stores and accumulation in muscle lactate levels. AST and the W-A significantly reduced skeletal muscle lactate (*p* < 0.001). Oxymetholone significantly increased the content of skeletal muscle glycogen (*p* < 0.05), and the W-A showed an increasing trend (*p* = 0.068). Succinate dehydrogenase (SDH) activity is a performance of mitochondrial oxidative capacity [41]. A comparison between young and old rats showed that SDH activity decreases in the muscles of old rats [42]. All treatment groups showed higher SDH levels, and the Oxy group showed significant differences (*p* < 0.05) (Figure 8A–C). PGC-1α plays a key role in the regulation of glucose catabolism, mitochondrial biogenesis, and oxidative metabolism [43]. The overexpression of Foxo3 decreases glucose uptake and affects glycogen synthesis, and many studies have reported that Foxo3 was upregulated by dexamethasone [44,45]. The W-A significantly reduced the expression of Foxo3 compared with the Sar group (*p* < 0.01), significantly increased PGC-1α expression (*p* < 0.05), and improved skeletal muscle metabolism better compared with other groups (Figure 8D,E). It indicated that the W-A could improve skeletal muscle atrophy by further improving skeletal muscle glycometabolism.

## 4. Discussion

In this study, the W-A nanoemulsions with better astaxanthin bioaccessibility as well as odor masking for stable storage at 4 °C were prepared by adjusting the astaxanthin concentration and the oil/water ratio. The optimal concentration of astaxanthin was determined to be 30 mg/mL, with an oil/water ratio of 1:5. Higher encapsulation rates and storage stability were demonstrated under these conditions. The low oil/water ratio might lead to degradation or loss of carotenoids due to the higher aqueous phase, which made acoustic cavitation during homogenization more susceptible to the formation of free radicals [22,46]. A high oil/water ratio might result in insufficient AST encapsulated capacity of WPI and thus a lower encapsulation rate. The size of the produced emulsion was around 110 nm (Figure 3B), which was similar to previous studies, and emulsions of this particle size improved the apparent permeability coefficient (P_app_) of Caco-2 cells to astaxanthin [18]. Interestingly, WPI performed best across all emulsifiers [22]. These results suggested that WPI was a good carrier for astaxanthin due to its emulsifying properties. This might be related to the binding of astaxanthin to WPI. As reported, the tight insertion of AST into the hydrophobic pockets of proteins enhanced physical stability [14].

The bioaccessibility of astaxanthin in the W-A was increased by 16-fold compared to astaxanthin (Figure 4D), and this might help astaxanthin work better. An increasing number of studies have focused on the bioavailability and bioaccessibility of actives in relation to their actual activity, and a variety of measures have been proposed to improve the two parameters of actives, such as nanoemulsions, Pickering emulsions, and lipid nanoparticles [47]. Our previous study found that astaxanthin-loaded emulsions were associated with further improvement in obesity [48]. It has also been shown that patients with celiac disease and Crohn’s disease had 37% lower levels of macular carotenoids compared to controls, even though plasma carotenoid concentrations were normal [49]. Therefore, the actual absorption of active substances is important for health and emulsions may contribute to better utilization through increased bioavailability and bioaccessibility. Besides, the odor of astaxanthin is another factor that troubles its application. Encapsulation of astaxanthin is an effective way to mask bad odors. Wanjun Zhao et al. found that the use of whey protein and xanthan gum to make an oleogel from Antarctic krill oil was effective in masking the off-odors in Antarctic krill oil [28,50]. In this study, astaxanthin emulsion prepared with WPI masked the off-flavor of astaxanthin and added some aroma substances with milky and fruity flavors.

For skeletal muscle atrophy, common treatments include exercise, protein supplementation, and antioxidant therapy, which is gaining attention. In this study, the W-A group had a better improvement in the mass of gastrocnemius, tibialis anterior, and soleus muscles than the WPI and AST groups, showing a better skeletal muscle atrophy relief effect. MuRF1 is a skeletal muscle ubiquitin protein ligase that promotes the ubiquitinated degradation of skeletal muscle. MyoD1 regulates myoblast differentiation and promotes muscle production. P70S6K, a downstream target protein of the skeletal myogenesis signaling pathway (mTOR), promotes muscle synthesis [51]. Various substances like saikosaponin and fenofibrate have been reported to improve skeletal muscle atrophy by affecting related proteins [52,53]. In our study, we found that WPI demonstrated good effects in restoring MyoD1 and reducing 4EBP1, suggesting that WPI was helpful in promoting skeletal muscle regeneration compared with AST, which might be related to the physiological function of leucine. Leucine is a branched-chain amino acid found in WPI. WPI and leucine-enriched essential amino acid (L-EAA) have been reported to increase muscle mass and improve muscle strength and function [54,55,56]. They are reported to be essential for proper muscle synthesis [56,57,58].

Astaxanthin also showed a tendency to promote skeletal muscle synthesis and reduce skeletal muscle degradation. Our present research showed that astaxanthin ameliorated cancer cachexia caused by C26 cells via this pathway [10]. In addition, three antioxidants, including astaxanthin, β-carotene, and resveratrol, have been reported to mediate the increase in mouse flounder muscle mass through mTOR and its downstream P70S6K [59]. Taken together, astaxanthin and WPI showed good potential for both synthesis and degradation of skeletal muscle, with W-A showing the best results, demonstrating the good synergistic potential of both.

Both WPI and AST suppressed mitochondrial autophagy as well as improved glycometabolism, with the W-A exhibiting an even greater effect. This might be due to the further improvement of mitochondrial function by the W-A, which showed suppressed mitochondrial autophagy through reduced BNIP3 expression and the ratio of Lc3B-ii/i. Astaxanthin has been reported to have a favorable effect on alleviating mitochondrial autophagy [10]. In this study, WPI demonstrated an effect close to that of astaxanthin, and this synergistic effect might lead to a better effect of the W-A. In addition, the W-A significantly increased the expression of PGC-1α, which might be related to mitochondrial function and energy metabolism, because PGC-1α, as a master regulator of mitochondrial biogenesis, regulated genes involved in cellular energy metabolism [60]. It was reported that astaxanthin preserves mitochondrial biogenesis by increasing the expression of PGC-1α in the flounder muscle of hindlimb-unloaded mice [61]. Our previous studies have also individually demonstrated the excellent modulation of glycolipid metabolism in skeletal muscle by astaxanthin [11]. There are also a few reports confirming the effect of WPI in restoring mitochondrial PGC-1α expression [62] and restoring mRNA expression of adipose tissue PGC-1α, albeit with limited effect on protein expression [63]. In this study, AST was slightly more effective than WPI in restoring PGC-1α by western blotting, and the W-A showed even greater enhancement (*p* < 0.01), which might account for the enhanced effect of AST due to the higher bioaccessibility.

Besides, PGC-1α responds to environmental and intracellular conditions like ROS and is regulated by SIRT1/3, TFAM, and AMPK, and the modulatory effects of AST on AMPK-related signaling pathways have also been reported [64]. Therefore, the regulation of PGC-related upstream signaling factors by astaxanthin and the regulation of ROS may jointly mediate the relevant effects.

In conclusion, this study constructed astaxanthin-loaded WPI nanoemulsions and investigated the effects on improving skeletal muscle atrophy, which provided a new reference for the precise nutritional design of astaxanthin and WPI. However, the study still has some limitations: (1) In addition to the mechanisms mentioned in the study, whether and how the antioxidant, anti-inflammatory, and gut microbiota-regulating abilities of astaxanthin and WPI are involved in the regulation of skeletal muscle atrophy need to be further investigated; (2) The mechanism of how WPI and astaxanthin finely synergize to regulate skeletal muscle atrophy needs to be further clarified.

## 5. Conclusions

In this study, astaxanthin-loaded WPI nanoemulsions with good bioaccessibility as well as undesirable flavor masking effects were prepared and characterized. The W-A demonstrated further improvement in dexamethasone-induced skeletal muscle atrophy by promoting skeletal myogenesis and inhibiting mitochondrial autophagy. In addition to this, the W-A further improved the glycometabolism of skeletal muscle. The W-A nanoemulsion shows promising application value in alleviating skeletal muscle atrophy, which contributes to the further intensive application of astaxanthin and WPI in precision nutrition. This study enriches the delivery modes of astaxanthin to meet the current diversified consumer needs and also proposes a strategy to rationally design the delivery system according to the needs.

## Figures and Tables

**Table 1:** Primer sequences.

Gene	Forward (5′–3′)	Reverse (5′–3′)
MuRF1	ACCACAGAGGGTAAAGAAGAACA	GCAGAGAGAAGACACACTTCCC
MAFbx	ACAAAGGAAGTACGAAGGAGCG	GGCAGTCGAGAAGTCCAGTC
P70S6K	TGTCAGCCCAGTCAAATTCTCTCC	ACATCCATCTGCTCTATCCCACTTG
4EBP1	ACCCAGTCCTGCTCCTCACTC	CTCGGTATAGACAGAGGCACAAGG
BNIP3	TTCTCACTGTGACAGCCCAC	TCTTCCTCAGACAGAGTGCT

## Data Availability

The original contributions presented in this study are included in the article. Further inquiries can be directed to the corresponding author.
